# Automated Insulin Delivery Systems and Glucose Management in Children and Adolescents With Type 1 Diabetes: A Systematic Review and Meta-Analysis

**DOI:** 10.1001/jamapediatrics.2025.2740

**Published:** 2025-09-08

**Authors:** Hannah Steiman de Visser, Seerat Waraich, Manik Chhabra, Jennifer Yamamoto, Ian Zenlea, Nicole Askin, Rasheda Rabbani, Jonathan McGavock

**Affiliations:** 1 Diabetes Research Envisioned and Accomplished in Manitoba (DREAM) Research Theme, Children’s Hospital Research Institute of Manitoba, Winnipeg, Canada; 2 Department of Pediatrics and Child Health, Rady Faculty of Health Sciences College of Medicine, University of Manitoba, Winnipeg, Canada; 3 Faculty of Health Sciences, Queens University, Kingston, Ontario, Canada; 4 Department of Pharmacology and Therapeutics, Rady Faculty of Health Sciences, University of Manitoba, Winnipeg, Canada; 5 Section of Adult Endocrinology, Department of Internal Medicine, Rady Faculty of Health Sciences, University of Manitoba, Winnipeg, Canada; 6 Institute for Better Health, Trillium Health Partners, Mississauga, Ontario, Canada; 7 Diabetes Action Canada SPOR Network, Toronto, Ontario, Canada; 8 Neil John Maclean Library, Rady Faculty of Health Sciences, University of Manitoba, Winnipeg, Canada; 9 George & Fay Yee Centre for Healthcare Innovation (CHI), Rady Faculty of Health Sciences, University of Manitoba, Winnipeg, Manitoba, Canada

## Abstract

**Importance:**

Youth living with type 1 diabetes (T1D) are increasingly choosing automated insulin delivery (AID) systems to manage their blood glucose. Few systematic reviews meta-analyzing results from randomized clinical trials (RCTs) are available to guide decision-making.

**Objective:**

To study the association of prolonged AID system use in an outpatient setting with measures of glucose management and quality of life in youth with T1D.

**Data Sources:**

MEDLINE, Embase, CINAHL, and Cochrane Central were searched from January 2017 to March 2025 to identify eligible RCTs.

**Study Selection:**

Two reviewers independently performed literature screening, data extraction, and quality assessment. Included in the analysis were RCTs of youth aged 6 to 18 years with T1D that assessed the efficacy of AID systems in outpatient settings longer than 48 hours compared with any other insulin regimen.

**Data Extraction and Synthesis:**

Two reviewers performed data extraction and quality assessment independently and reported using the Preferred Reporting Items for Systematic Reviews and Meta-Analyses (PRISMA) and PRISMA literature search extension guidelines. Random-effects meta-analysis models were used to estimate the pooled measures of efficacy as a mean difference (MD) with 95% CIs for outcomes measures.

**Main Outcomes and Measures:**

The 2 primary outcome measures were time in range (TIR) and glycated hemoglobin (HbA_1c_).

**Results:**

Of 2363 citations retrieved, 11 RCTs (n = 901 participants) with measures of HbA_1c_ and 10 RCTs (n = 786 participants) with measures of TIR were included. RCTs tested interventions lasting a mean (SD) of 31 (26) weeks on youth with a median age of 12 years (range, 10.8-15.9 years); 51% were female, mean (SD) HbA_1c_ level was 8.4% (1.1%), and mean (SD) TIR was 51% (9%). Random-effects models revealed that, compared with any insulin regimen, HbA_1c_ level was reduced −0.41% (95% CI, −0.58% to −0.25%; *I*^2^ = 39%), whereas TIR increased 11.5% (95% CI, 9.3%-13.7%; *I*^2^ = 23%) with nighttime TIR increasing 19.7% (95% CI, 17.0%-22.4%; *I*^2^ = 36%). Random-effects models also revealed that AID use was associated with reduced time spent in hypoglycemia (<3.9 mml/L; MD = −0.32%; 95% CI, −0.60% to −0.03%; *I*^2^ = 18%) and hyperglycemia (>10 mmol/L; MD = −10.8%; 95% CI, −14.4% to −7.2%; *I*^2^ = 55%), particularly during the night (MD = −14.4%; 95% CI, −19.9% to −8.9%; *I*^2^ = 79%) compared with any insulin regimen. There were no differences in adverse events between study arms. Only 2 studies reported changes in QOL.

**Conclusions and Relevance:**

This systematic review and meta-analysis found that compared with any other insulin regimen, use of AID systems by youth with T1D was associated with clinically meaningful improvements in multiple measures of glucose management, including the risk of both hyperglycemia and hypoglycemia, without increasing the risk of adverse events. More data are needed on the efficacy of AID systems on patient report outcomes.

## Key Points

**Question:**

In children and adolescents living with type 1 diabetes, are automated insulin delivery (AID) systems used in an outpatient setting associated with improvements in measures of glucose management and quality of life compared with standard care?

**Findings:**

This systematic review and meta-analysis of 11 trials and 901 patients with type 1 diabetes found that, compared with standard care, AIDs use for more than 6 months was associated with clinically meaningful improvements multiple measures of glucose management, particularly during the nighttime without increasing adverse events.

**Meaning:**

Results suggest that AIDs are an effective way to improve measures of glucose management in children and adolescents with type 1 diabetes; however, the effect of AIDs on quality of life remains unclear.

## Teaser

This systematic review and meta-analysis investigates if, compared with standard care, automated insulin delivery systems used in an outpatient setting improve measures of glucose management and quality of life in children and adolescents with type 1 diabetes.

## Introduction

Children and adolescents (youth) living with type 1 diabetes have several options for managing their blood glucose.^1,2,3^ Recent technological advances in continuous glucose monitoring and continuous subcutaneous insulin infusion pumps have reduced the burden of glucose self-management while improving glucose management and lowering the risk of hypoglycemic events.^3,4,5^ The most recent advancement in glucose management integrates these 2 technologies into an automated insulin delivery (AID) system, with an algorithm that titrates insulin delivery according to real-time glucose levels.^6,7,8,9,10^ As more people living with type 1 diabetes adopt AID systems^11^ and national health systems make decisions about covering them for diabetes care,^12,13^ a systematic analysis of the efficacy of prolonged AID use in an outpatient setting on measures of glucose management in youth is warranted.

A recent clinical practice guideline^14^ suggests there is strong evidence that AID systems are efficacious for improving measures of glucose management; however, the systematic reviews that inform this guideline have methodological limitations that limit their translation to clinical practice.^15,16,17,18,19,20^ The meta-analyses of clinical trials that informed this guideline suggest that AID system use increases time spent with glucose in the range of 3.9 and 10.0 mmol/L (to convert to milligrams per deciliter, divide by 0.0555), referred to as *time in range* (TIR), while simultaneously reducing glycated hemoglobin (HbA_1c_) level. However, several key gaps in these reviews limit their external validity and potential application into a clinical setting. First, most reviews included short-term (<7 days) safety trials,^21,22^ some within diabetes camp settings,^23,24^ crossover trials that included prolonged inpatient time under supervised conditions^25^ or, quasi-experimental and observational studies of AID systems.^17^ Second, randomized clinical trials (RCTs) compared AID systems with a variety of glucose management strategies ranging from sensor-augmented pump therapy to multiple daily injections with and without continuous glucose monitoring. Further, recent RCTs of long-term, outpatient AID use^7,23,26,27,28,29^ suggest that improvements in TIR are greatest at night; however, no systematic reviews, to our knowledge, have empirically compared effects of AID systems on glucose management between the nighttime and daytime. Furthermore, no systematic reviews, to our knowledge, have summarized the effects AID systems on patient-reported outcome measures for youth living with type 1 diabetes.^30,31^ Lastly and to our knowledge, none of the reviews published to date summarized the certainty of the evidence using the Grading of Recommendations Assessment, Development, and Evaluation (GRADE) reporting guidelines for main outcome measures and adverse events.^32,33^ To overcome limitations of previous reviews, the main objective of the current systematic review and meta-analysis was to determine the size and precision of the association of AID use over a prolonged period in an outpatient setting with measures of glucose management stratified by time of day and quality of life in youth living with type 1 diabetes.

## Methods

### Eligibility Criteria and Outcomes

This systematic review and meta-analysis was registered with Prospero (CRD42024555186) and reported according to the Preferred Reporting Items for Systematic Reviews and Meta-analyses (PRISMA) 2020 reporting guidelines.^34,35^ The search was restricted to RCTs that enrolled at least 1 youth aged 6 to 18 years living with type 1 diabetes, had used any type of AID system as an intervention to manage blood glucose for longer than 48 hours, and included a comparison arm. The primary outcomes included mean TIR (percentage of time spent in blood glucose levels above 3.9 mmol/L and below 10 mmol/L), and mean HbA_1c_ levels. Secondary outcomes included mean time spent in hypoglycemia (≤3.9 mmol/L) and hyperglycemia (≥10 mmol/L) during the day and nighttime as well as measures of glucose variability and patient reported outcomes.

### Data Sources and Search Strategy

An experienced health sciences librarian (N.A.) searched MEDLINE (Ovid), Embase (Ovid), CINAHL with full text (EBSCO), and Cochrane Central (Ovid) from January 1, 2017, to June 5, 2024, originally and updated through to March 5, 2025, for articles reporting results from RCTs of AID use in children and adolescents living with type 1 diabetes. The search strategy was peer-reviewed by another librarian using the Peer Review of Electronic Search Strategies checklist.^36^ The search results were restricted to English language studies only. International Clinical Trials Registry Platform, ClinicalTrials.gov, gray literature, and manual searches were also performed to identify potential studies. All search results were imported into Rayyan for deduplication and screening. The full search strategy and result counts for each database are provided in eTables 1 to 8 in Supplement 1.

### Study Selection, Data Extraction, and Quality Assessment

Two reviewers (H.S.D., S.W.) performed title and abstract screening, full-text screening, data extraction, and quality assessment independently. Any reviewer disagreements were resolved through discussion with a third reviewer (J.M.). Before the full screen, a preliminary screen of 30 abstracts was conducted to determine an agreement score. After reaching an agreement score of 93.3% (28 of 30 abstracts in agreement), indicating high agreement between the reviewers, the full screening process proceeded. No automation tools were used. Study investigators were contacted to obtain or confirm unclear or missing data if necessary. All available data at all time points and comparisons were collected for each outcome. No automation tools were used for any step while conducting this systematic review. Data were extracted for trial characteristics, study participant information, intervention and control arm information, outcome measures, study withdrawal, and adverse events. The Cochrane Risk of Bias tool, version 1, was used to assess the internal validity of the included RCTs.^37^

### Data Synthesis

Data for each meta-analysis were determined based on the availability of the outcome measure and information for subgroup analyses. Control arms subgroups were classified as sensor augmented pump or other, which included a range of self-management strategies. Studies with missing data for outcomes analyses were excluded. Meta-analysis results are presented as forest plots with summary statistics for each outcome, and where possible, outcomes were displayed for nighttime and daytime data collection separately.

### Assessing Certainty of the Evidence

We used the GRADE reporting recommendation guidelines to assess the certainty of the evidence for each outcome of interest in each of the following domains: (1) risk of bias, (2) imprecision, (3) inconsistency, (4) indirectness, and (5) publication bias for all of the primary and secondary outcomes. We used a clinically meaningful difference of 5% for measures of TIR,^38^ and time in hyperglycemia, 1% for time in hypoglycemia^38^ and 0.5% for HbA_1c_.^39^

### Inclusion of Persons With Lived Experience in this Study

Patient coresearchers were involved in the design, delivery, and interpretation of a clinical trial were invited to participate in this review. Patient coresearchers with lived experience helped in the design aspects of the analysis, interpretation of findings, and preparation of this article.

### Statistical Analysis

When at least 2 studies of similar populations, methods, and outcomes were available, inverse variance–weighted random-effects meta-analyses were performed to calculate mean differences (MDs) with 95% CIs between intervention and control arms. The range of differences and the inconsistency index (*I*^2^) were used to quantify heterogeneity among studies. Subgroup analyses were performed to determine if differences in outcomes were influenced by the timing of the effects (nighttime vs daytime outcomes), duration of the intervention, and the glucose management strategy of the control condition. Risk differences were used to compare differences in adverse events between study arms. Funnel plots were used to investigate publication bias using the Egger test, and visual inspection was used to assess plot asymmetry for meta-analyses of the 2 primary outcome measures. As no trials reported results for sex and gender subgroups, we were unable to conduct sex and gender-based analyses. In those studies that reported race and ethnicity, participants self-identified the following races and ethnicities: Black, Hispanic, Maori, multiracial, Native American, and White. The meta-analyses were conducted using the general meta and metafor packages in RStudio,^40^ version 4.3.0 (R Project for Statistical Computing).

## Results

PRISMA flowchart describes the complete study selection process (eFigure 1 in Supplement 1). Of 2363 identified citations, 11 unique RCTs, including 902 youth with type 1 diabetes (51% female; 49% male; median age, 12.0 years; range 10.8-15.9 years), were included in the analyses.^7,8,9,28,29,41,42,43,44,45,46^ The median duration of diabetes among youth in the identified trials was 6.1 years (range, 5-7 years; n = 3 trials). Participants self-reported the following races and ethnicities: 4% Black, 3% Hispanic, 2% Maori, 5% multiracial, 0.3% Native American, and 82% White (range, 64%-94%; n = 8 trials). Of the 901 youth enrolled into 11 RCTs, 524 were randomized to an AID system, and 377 were randomized to controls (n = 786: 458 in AID arm and 328 in control arm for trials focused on TIR). Trials were conducted in the US (5 of 11),^8,9,44,45,46^ UK (1 of 11),^7^ Oceania (2 of 11),^41,42^ and across multiple sites including but not limited to the US and the UK (3 of 11).^28,29,43^ Of the 11 trials included, 1 was industry funded, and governmental agencies funded the remaining 10. Trials tested interventions of AID system use for a mean (SD) duration of 31 (26) weeks, ranging from 12 to 104 weeks (Table 1). Baseline characteristics of children and adolescents randomized to AID and control are provided in the eTable 9 in Supplement 1.

**Table 1.** Intervention and Trial Characteristics of Randomized Trials Included in the Meta-Analysis

Study	Centers, No.	Sample size, No.	Age, y	Pump (manufacturer)	CGM	Algorithm	Duration, wk	Control	Funding	Primary outcome
Tauschmann et al,^28^ 2018	6	23	6-13	Mini-Med 670G (Medtronic)	Medtronic Enlite 3	FlorenceM	12	SAP	JDRF, NIHR, WT	TIR
Breton et al,^8^ 2020	4	101	6-12	t:slim X2 (Tandem)	Dexcom G6	Control-IQ	16	SAP	Tandem + NIDDK	TIR
Abraham et al,^41^ 2021	5	110	12-18	MiniMed 670G (Medtronic)	Guardian Sensor 3	SmartGuard	26	CSII or MDI	JDRF + Australian NHMRC	TIR
Isganaitis et al,^44^ 2021	7	48	14-18	t:slim X2 (Tandem)	Dexcom G6	Control-IQ	24	SAP	NIDDK	TIR
Burnside et al,^42^ 2022	4	48	7-15	DANA-I (Advanced Therapeutics UK)	Dexcom G6	OpenAPS 0.7.0	24	SAP	HRCNZ	TIR
Reiss et al,^46^ 2022	5	42	14-17	MiniMed 670G (Medtronic)	Dexcom G5/6	NA	24	MDI or SAP	NICHD JDRF	Gray matter metrics
Ware et al,^29^ 2022	12	133	6-18	Dana Diabecare RS (Advanced Therapeutics UK)	Freestyle Libre	FlorenceM or CamAPS	24	CSII	NIDDK	HbA_1c_
Boughton et al,^7^ 2022	7	97	10-17	Tandem t:slim X2 (Tandem)	Dexcom G6	CamAPS	104	MDI	NIHCR	C-peptide AUC
Messer et al,^45^ 2022	10	165	6-17	iLet pump (Beta Bionics)	Dexcom G6	Bionic Pancreas	13	Dexcom G6	NIDDK	HbA_1c_
McVean et al,^9^ 2023	6	108	7-17	t:slim X2 (Tandem) or MiniMed 670G (Medtronic)	Dexcom G6	Control-IQ or SmartGuard	52	Dexcom G6	JDRF	C-peptide AUC
Garg et al,^43^ 2023	23	59	2-17	MiniMed 670G (Medtronic)	Guardian Sensor 3	SmartGuard	24	SAP	Medtronic	HbA_1c_

Abbreviations: AUC, area under the curve; CGM, continuous glucose monitor; CSII, continuous subcutaneous insulin infusion; HbA_1c_, glycated hemoglobin; HRCNZ, Health Research Council of New Zealand; JDRF, Juvenile Diabetes Research Foundation (now Breakthrough T1D); MDI, multiple daily injections; NA, not available/reported; NHMRC, National Health and Medical Research Council (Australia); NICHD, Eunice Kennedy Shriver National Institute of Child Health and Human Development; NIDDK, National Institute of Diabetes and Digestive and Kidney Diseases; NIHCR, National Institute for Health Care Reform; SAP, sensor augmented pump; TIR, time in range; WT, Welcome Trust.

Three trials studied Cambridge APS (CamDiab) and/or Florence M (Microsoft) algorithm,^7,28,29^ 3 studied a SmartGuard (Medtronic) algorithm^41,43,46^ linked to a Medtronic 670 pump, 2 studies used a Tandem t:slim X2 (Tandem Diabetes) system,^8,44^ 1 study used both Medtronic and Tandem t:slim X2 systems,^9^ 1 examined an open source Do-It-Yourself algorithm,^42^ and, 1 used an algorithm integrating an iLet insulin pump (Beta Bionics) with a Dexcom G6 continuous glucose monitor^45^ (Table 1). At baseline, the mean (SD) HbA_1c_ level and TIR were 8.4% (1.1%; to convert to proportion of total hemoglobin, multiply by 0.01; n = 11 trials^7,8,9,28,29,41,42,43,44,45,46^; n = 938 participants) and 51% (9%; n = 10 trials^7,8,9,29,41,42,43,44,45,46^; n = 786 participants), respectively (eTable 9 in Supplement 1). AID systems were in use, on average, 87% of the time (range, 75%-96%) for the duration of the intervention (n = 8 trials^7,8,9,29,41,42,44,45^).

### Primary Outcomes

**Figure 1.**
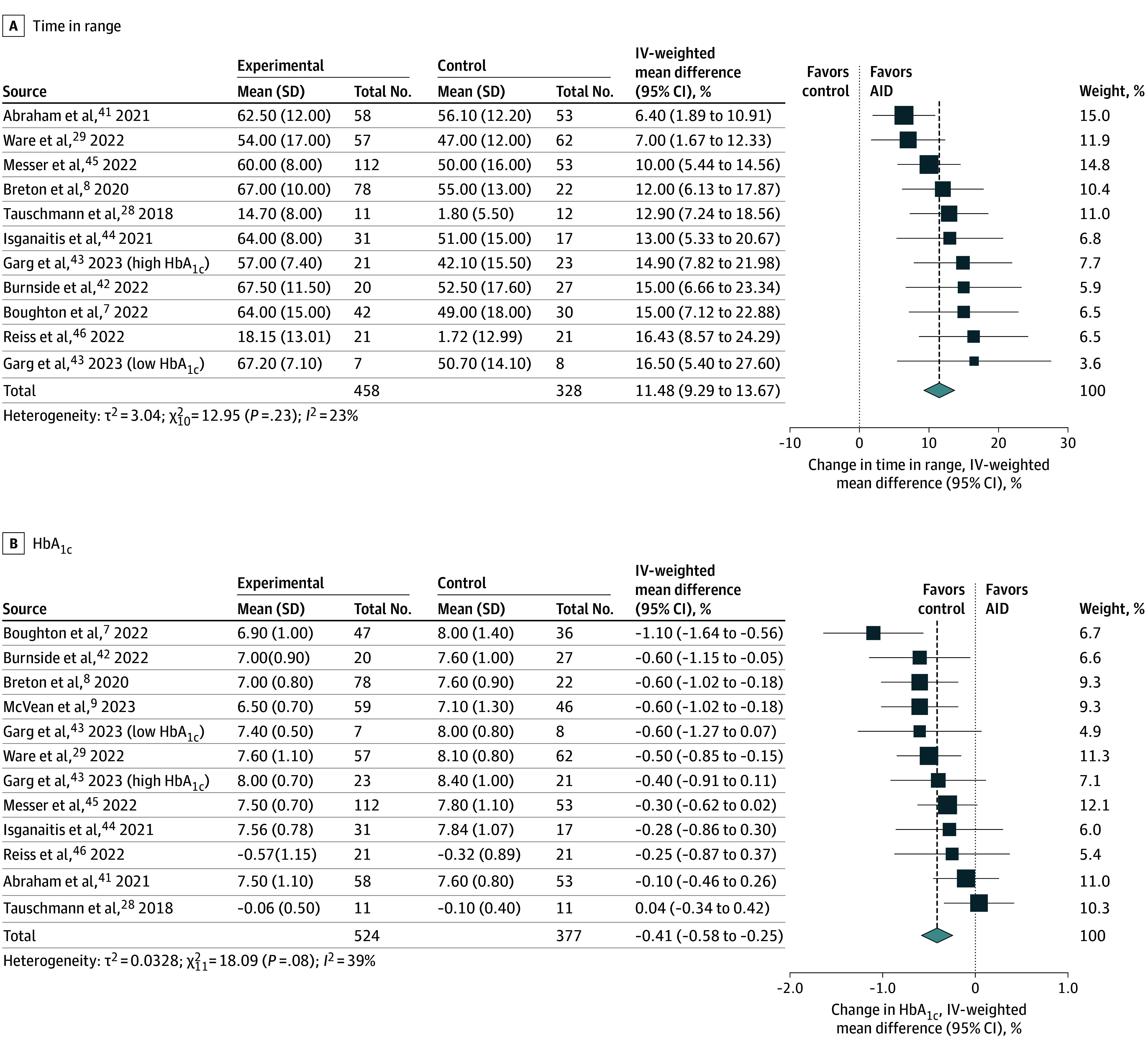
Association of Automated Insulin Delivery (AID) With Measures of Glucose Control HbA_1c_ indicates glycated hemoglobin; IV, inverse variance.

Compared with youth randomized to any insulin regimen, those randomized to an AID system had their TIR increase an average of 11.5% (95% CI, 9.3%-13.7%; *I*^2^ = 23%; n = 10 trials^7,8,28,29,41,42,43,44,45,46^; n = 786 participants) (Figure 1A), and their HbA_1c_ level was reduced an average of −0.41% (95% CI, −0.58% to −0.25%; *I*^2^ = 39%; n = 11 trials^7,8,9,28,29,41,42,43,44,45,46^; n = 901 participants) (Figure 1B).

### Secondary Outcomes

**Figure 2.**
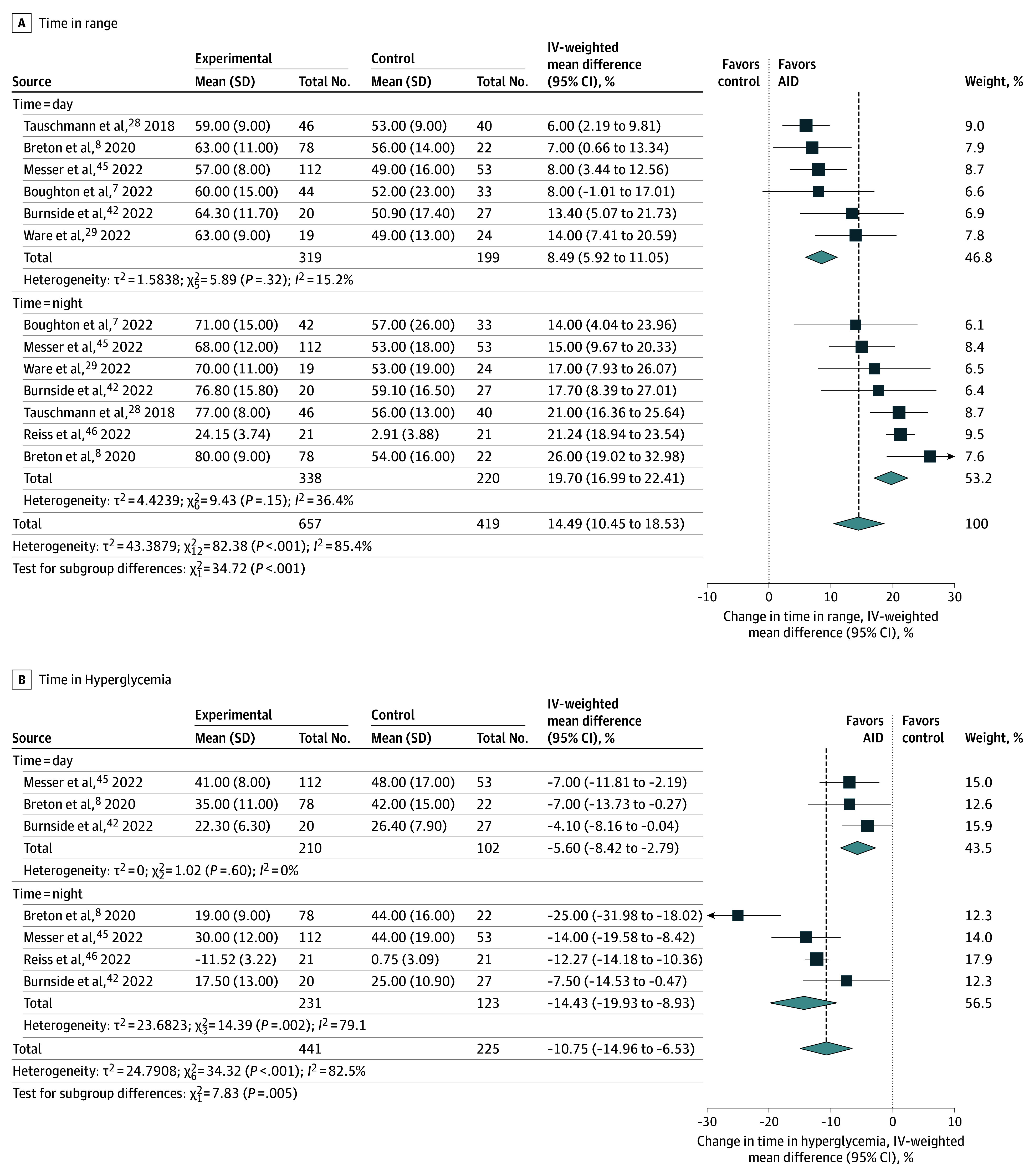
Association of Automated Insulin Delivery (AID) With Measures of Time in Range IV indicates inverse variance.

When continuous glucose monitor outcome data were stratified by time of day, improvements in TIR were greater during nighttime data collection (MD = +19.7%; 95% CI, 17.0%-22.4%; *I*^2^ = 36%; n = 7 trials^7,8,28,29,42,45,46^; n = 558 participants) compared with daytime data collection (MD = +8.5%; 95% CI, 5.9%-11.1%; *I*^2^ = 15%; n = 6 trials^7,8,28,29,42,45^; n = 518 participants) (Figure 2A). The time spent in hypoglycemia (<3.9 mml/L; MD = −0.32%; 95% CI, −0.60 to −0.03%; *I*^2^ = 18%; n = 7 trials^7,8,28,29,41,42,43,44,45,46^; n = 580 participants) and hyperglycemia (>10 mmol/L; MD = −10.8%; 95% CI −14.4% to −7.2%; *I*^2^ = 55%; n = 7 trials^7,8,29,41,43,44,45^; n = 674 youth) were both reduced with the use of an AID system compared with control glucose management (Figure 2B and eFigure 5 in Supplement 1, respectively). Reductions in time spent in hyperglycemia were larger at night than during the day (nighttime MD = −14.4%; 95% CI, −19.9% to −8.9%; *I*^2^ = 79%; n = 4 trials^8,42,45,46^; n = 354 participants vs daytime MD = −5.6%; 95% CI, −8.4% to −2.8%; *I*^2^ = 0%; n = 3 trials^8,42,45^; n = 312 participants) (Figure 2B). Similarly, reductions in time spent in hypoglycemia were also larger at night than during the day (nighttime MD = −0.62%; 95% CI, −1.02% to −0.23%; *I*^2^ = 13%; n = 5 trials^7,8,29,42,45^; n = 430 participants vs daytime MD = −0.30%; 95% CI, −0.72% to +0.12%; *I*^2^ = 0%; n = 4 trials^8,29,42,45^; n = 355 participants) (eFigure 5 in Supplement 1).

Most trials reported changes in at least 1 measure of glucose variability.^7,8,29,41,42,43,45,46^ The use of an AID system was associated with a reduction in SD of glucose (MD = −4.68; 95% CI, −8.14 to −1.22; *I*^2^ = 71%; n = 7 trials^7,8,29,41,42,43,45^; n = 602 participants) (eFigure 2A in Supplement 1) but not with a difference in the glucose coefficient of variation compared with control glucose management (MD = −0.41; 95% CI, −2.20 to +1.38; *I*^2^ = 81%; n = 8 trials^7,8,29,41,42,43,45,46^; n = 644 participants) (eFigure 2B in Supplement 1)

### Subgroup Analyses

Subgroup analyses are presented in Table 2. Trials of AID system use greater than 6 months were associated with greater reductions in HbA_1c_ level (MD = −0.47%; 95% CI, −0.66; −0.28%; *I*^2^ = 28%; n = 8 trials; n = 614 participants) than trials of interventions lasting less than 6 months (MD = −0.28%; 95% CI, −0.62; +0.06%; *I*^2^ = 28%; n = 3 trials; n = 287 participants). No differences in the improvement in TIR were observed between trials of AID use less than 6 months (MD = +11.37; 95% CI, 8.33; 14.41%; *I*^2^ = 0%; n = 3 trials; n = 288 participants) and those greater than 6 months (MD = +11.99; 95% CI, 8.70%-15.29%; *I*^2^ = 43%; n = 8 trials; n = 498 participants). The duration of trial did not affect the magnitude of the improvements in time spent in hyperglycemia or in hypoglycemia. Similarly, the efficacy of AID systems was not influenced by the comparator.

**Table 2.** Results From Subgroup Analyses for Selected Measures of Glucose Management

Outcome	Trial, No.	*I*^2^, %	Participant, No.		Mean difference (95% CI)
**AID arm**	**Control arm**
**HbA_1c_ (%)**					
Duration of trials					
< 6 mo	9	28	323	291	−0.47 (−0.66 to −0.28)
≥ 6 mo	3	60	201	86	−0.28 (−0.62 to −0.06)
Type of control					
SAP	6	27	170	106	−0.37 (−0.61 to −0.13)
Other	6	54	354	271	−0.45 (−0.70 to −0.20)
**Time below 70 mg/dL (%)**					
Duration of trials					
< 6 mo	6	41	157	158	−0.26 (−1.11 to 0.60)
≥ 6 mo	2	0	190	75	−0.32 (−0.55 to −0.09)
Type of control					
SAP	5	0	157	97	−0.35 (−0.64 to −0.06)
Other	3	60	190	136	0.38 (−0.88 to 1.63)
**Time above 180 mg/dL (%)**					
Duration of trials					
< 6 mo	6	66	216	193	−11.27 (−16.57 to −5.96)
≥ 6 mo	2	0	190	75	−10.11 (−13.93 to −6.30)
Type of control					
SAP	4	0	137	70	−12.57 (−16.62 to −8.52)
Other	4	77	269	198	−9.66 (−15.66 to −3.67)
**Time in range 70-180 mg/dL (%)**					
<6 mo	8	43	257	241	11.99 (8.70 to 15.29)
≥6 mo	3	0	201	87	11.37 (8.33 to 14.41)
Type of control					
SAP	6	0	168	109	13.53 (10.64 to 16.42)
Other	5	47	290	219	10.00 (6.48 to 13.52)

Abbreviations: AID, automated insulin delivery; HbA_1c, _glycated hemoglobin; SAP, sensor augmented pump therapy.

SI conversion factor: To convert HbA_1c_ to proportion of total hemoglobin, multiply by 0.01.

### Adverse Events

**Figure 3.**
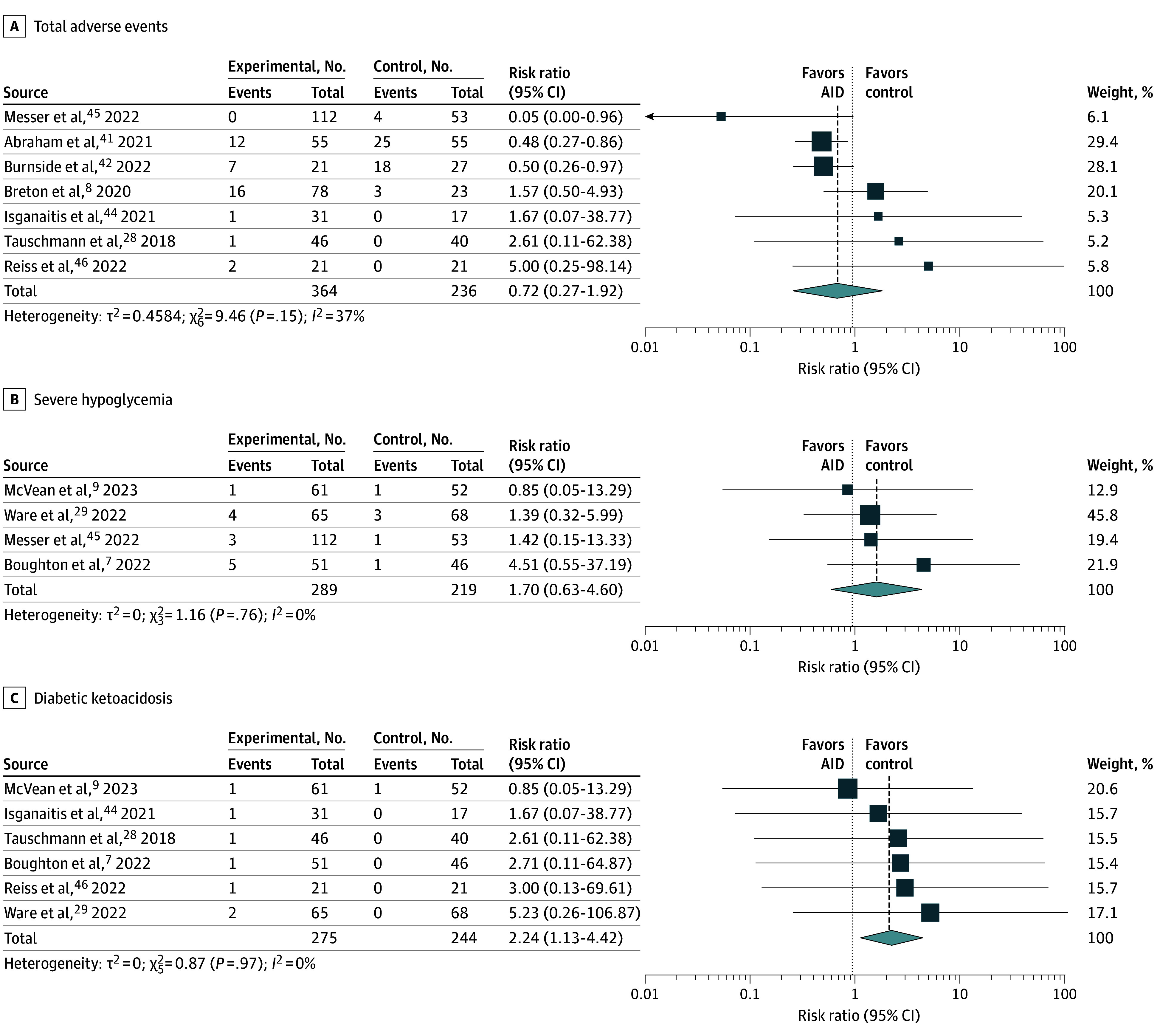
Association of Automated Insulin Devices (AIDs) With Adverse Events

Rates of severe hypoglycemia (n = 19 events from 841 youth) and diabetic ketoacidosis (n = 8 events from 841 youth) were rare (eTable 10 in Supplement 1). Of the 7 trials^8,28,41,42,44,45,46^ (n = 600 participants) that reported adverse events, the risk ratio between AID system use and control arm was 0.72 (95% CI, 0.27-1.92; *I*^2^ = 37%; n = 600 participants) (Figure 3A). Four trials^7,9,29,45^ reported rates of severe hypoglycemia, with a risk ratio of 1.70 (95% CI, 0.63-4.60; *I*^2^ = 0%; n = 508 participants) between AID system use and control arms (Figure 3B). Of the 8 cases of diabetic ketoacidosis reported among 519 youth from 6 trials^7,9,28,29,44,46^ (n = 519), 1 was observed in the control arm, and 7 were observed in the AID system arm, with an adverse event rate ratio of 2.24 (95% CI, 1.13-4.42; *I*^2^ = 0%; 6 trials; n = 519 participants) (Figure 3C).

### Risk of Bias and Publication Bias

The Cochrane risk of bias tool suggested most trials are at a high risk of bias due to the lack of blinding (eFigure 3 in Supplement 1). The risk was reduced in most trials with the application of blinded continuous glucose monitoring to capture measures of time in range. Several studies also failed to report outcome data using an intention-to-treat analysis, only reporting results for youth retained for follow-up measures. Funnel plots to estimate publication bias for the primary outcome measures are presented in eFigures 4A and B in Supplement 1. Egger tests suggested plot asymmetry for the outcome of time in range (T score = 4.3; *P* = .002) but not HbA_1c_ level (T score = −1.3; *P* = .22).

### Certainty of Evidence

A GRADE table summarizing the certainty of evidence for the main outcomes and adverse events is provided in eTable 11 in Supplement 1. For all measures of time in range and time in hyperglycemia (total, daytime, and nighttime) the observed effect size was 2 to 4 times higher than the minimally important difference and the risk of both bias and imprecision were low. For HbA_1c_ and time spent in hypoglycemia, the observed effect size and CIs overlapped with the minimally important difference. For adverse events, the risk of bias was low, but the risk of imprecision and inconsistency were high due to low rates of adverse events and wide CIs.

### Patient-Reported Outcomes

Only 2 trials reported quality of life,^28,41^ and 1 included a measure of the changes in diabetes treatment satisfaction.^43^ Neither were sufficient for meta-analysis. The patient coresearchers on our team identified gaps in several patient-reported outcomes associated with diabetes-related quality of life. These included changes in sleep quality for youth and their parents, diabetes distress, diabetes-specific burnout, and the effect of AID use on the perceived feasibility of participating in physical activity and, sport-related behaviors.

## Discussion

This systematic review and meta-analysis of RCTs found that outpatient AID system use for 12 weeks to 2 years was associated with a 0.4% reduction in HbA_1c_ level (from a preintervention value of approximately 8.5% HbA_1c_) and an 11% improvement in TIR (from a preintervention value of approximately 50%) for youth living with type 1 diabetes. Additionally, we found that the association of AID systems with measures of glucose management was greatest during the nighttime. These outcomes were not influenced by the duration of AID system use or the comparison glucose management strategy (ie, manual delivery, pump therapy alone or sensor augmented pump). The benefits of AID systems were also associated with reduced glucose variability without an increased risk of adverse events, including hypoglycemia, but may be associated with an increased risk for diabetic ketoacidosis. The certainty of the evidence for the association of AID with measures of glucose management was generally high, suggesting that these data could inform future clinical practice guidelines. Lastly, patient partners identified several gaps in patient-reported outcome measures in trials published to date, including quality of life, diabetes-related distress, and measures of sleep quality or sleep-related stress.

Advances in technology are rapidly improving the ability of youth living with type 1 diabetes to manage blood glucose.^5^ The most recent advancements, AID systems, are rapidly becoming a common strategy for managing blood glucose and minimizing the burden of manual self-management.^5,10^ Previous systematic reviews of clinical trials that included short-term safety and inpatient trials suggest that AID systems elicit clinically relevant improvements in TIR (10%-13%) and HbA_1c_ level (0.3%-0.5%) in youth with type 1 diabetes.^15,16,17^ The current systematic review supports previous evidence and expands it in several important ways. First, the 0.4% reduction in HbA_1c_ level and absolute approximately 11% improvement in TIR were nearly identical to effects reported in previous reviews,^15,16,17,18,19,20^ suggesting that the efficacy seen in short-tern safety trials is sustained during prolonged use in an outpatient setting. Second, the improvements in TIR are driven largely by improvements at night. Specifically, the improvement in TIR was over 2-fold greater (19% vs 9%) and the reduction in time in hyperglycemia was 3-fold greater (14% vs 5%) during nighttime than during the day. We also found that reductions in time spent in hypoglycemia were greatest during the nighttime, which may be related to reductions in glucose variability.^47^ Collectively, these data support the use of AID systems for improving glucose self-management for youth living with type 1 diabetes.

The adoption of new diabetes technology is intimately linked to youth’s perceived effectiveness with the device, balanced against the risk for adverse events.^10,48^ Qualitative studies and surveys suggest that youth with type 1 diabetes and their caregivers have positive experiences with the adoption of an AID system.^49,50,51^ The data presented here provide additional support for satisfaction and identified gaps in the current clinical trial evidence for patient-reported outcomes in this area. First, overall wear time for AID system use was over 85% among more than 500 youth treated for an average of 31 weeks, and dropout rates from the AID arm of the trials was less than 5%. These data are coupled with the observation that the overall rates of severe adverse events were rare. These data reinforce experiential data from youth and caregivers that the benefits of AID systems are evident without an increased risk of adverse events.^49,51^ Without sufficient clinical trial evidence, it remains unclear if AID use leads to meaningful improvements in key diabetes-specific patient-reported outcomes among youth living with type 1 diabetes. Including patient coresearchers and patient-reported outcomes in the design of future trials of AID use could quickly fill this knowledge gap.

### Limitations

The quality of a systematic review and meta-analysis depends on the quality of the trials included in it. Previous reviews of AID systems in youth with type 1 diabetes included data from nonrandomized trials, crossover trials, and short-term efficacy trials. We advanced the external validity of previous systematic reviews by restricting analyses to parallel-arm randomized trials of prolonged outpatient use. An additional limitation of the randomized trials in this area to date is the lack of diversity of among the youth randomized, particularly, there was limited participation from youth living with type 1 diabetes from Asian, Black, Hispanic, and Indigenous populations. This lack of racial and socioeconomic diversity of participants limits the external validity of these findings and should be addressed in future trials.

### Conclusions

This systematic review and meta-analysis found that prolonged AID use was associated with clinically meaningful improvements in glucose management in youth with type 1 diabetes. Data from a small number of events suggest that AID use may also be associated with an increased risk for diabetic ketoacidosis in a subset of youth living with type 1 diabetes, requiring further investigation. Future trials are needed to estimate the efficacy of AID systems on patient-reported outcomes.

## Notes

Supplement 1. **eTable 1.** Search Strategy and Results MEDLINE **eTable 2.** Search Strategy and Results Embase **eTable 3.** Search Strategy and Results Cochrane **eTable 4.** Search Strategy and Results CINAHL **eTable 5.** Search Strategy and Results MEDLINE (to March 11, 2025) **eTable 6.** Search Strategy and Results Embase (to March 11, 2025) **eTable 7.** Search Strategy and Results Cochrane (to March 11, 2025) **eTable 8.** Search Strategy and Results CINAHL (to March 11, 2025) **eTable 9.** Baseline Characteristics of Participants in AID Group vs Standard Diabetes Management Included in the Meta-Analysis **eTable 10.** Safety Outcomes Reported in Participants Included in the Meta-Analysis **eTable 11.** Certainty of Evidence for Observed Changes in Outcome Measures Using the GRADE Reporting Criteria **eFigure 1.** PRISMA Flow Chart **eFigure 2.** Effects of AID on Measures of Glucose Variability **eFigure 3.** Risk of Bias Metrics for Randomized Control Trials Included in the Meta-Analyses **eFigure 4.** Funnel Plots for Main Outcome Measures **eFigure 5.** Effects of AID on Measures of Time in Range

Supplement 2. **Nonauthor Collaborators.** TEAM Trial Patient Coresearchers

Supplement 3.
Data Sharing Statement.
