# Supplementary material for: Automated Insulin Delivery Systems and Glucose Management in Children and Adolescents With Type 1 Diabetes: A Systematic Review and Meta-Analysis
Source: JAMA Pediatr. 2025 Sep 8;179(11):1162–71. doi: 10.1001/jamapediatrics.2025.2740 (PMC12418225; doi:10.1001/jamapediatrics.2025.2740)
Supplement: Supplement 1. — eTable 1. Search Strategy and Results MEDLINE eTable 2. Search Strategy and Results Embase eTable 3. Search Strategy and Results Cochrane eTable 4. Search Strategy and Results CINAHL eTable 5. Search Strategy and Results MEDLINE (to March 11, 2025) eTable 6. Search Strategy and Results Embase (to March 11, 2025) eTable 7. Search Strategy and Results Cochrane (to March 11, 2025) eTable 8. Search Strategy and Results CINAHL (to March 11, 2025) eTable 9. Baseline Characteristics of Participants in AID Group vs Standard Diabetes Management Included in the Meta-Analysis eTable 10. Safety Outcomes Reported in Participants Included in the Meta-Analysis eTable 11. Certainty of Evidence for Observed Changes in Outcome Measures Using the GRADE Reporting Criteria eFigure 1. PRISMA Flow Chart eFigure 2. Effects of AID on Measures of Glucose Variability eFigure 3. Risk of Bias Metrics for Randomized Control Trials Included in the Meta-Analyses eFigure 4. Funnel Plots for Main Outcome Measures eFigure 5. Effects of AID on Measures of Time in Range [file jamapediatr-e252740-s001.pdf]

Supplementary Online Content

de Visser HS, Waraich S, Chhabra M, et al; TEAM Trial Patient Coresearchers. Automated insulin delivery systems and glucose management in children and adolescents with type 1 diabetes: a systematic review and meta-analysis. *JAMA Pediatr*. Published online September 8, 2025. doi:10.1001/jamapediatrics.2025.2740

eTable 1. Search Strategy and Results Medline

eTable 2. Search Strategy and Results Embase

eTable 3. Search Strategy and Results Cochrane

eTable 4. Search Strategy and Results CINAHL

eTable 5. Search Strategy and Results Medline (to March 11, 2025)

eTable 6. Search Strategy and Results Embase (to March 11, 2025)

eTable 7. Search Strategy and Results Cochrane (to March 11, 2025)

eTable 8. Search Strategy and Results CINAHL (to March 11, 2025)

eTable 9. Baseline Characteristics of Participants in AID Group vs Standard Diabetes Management Included in the Meta-Analysis

eTable 10. Safety Outcomes Reported in Participants Included in the Meta-Analysis

eTable 11. Certainty of Evidence for Observed Changes in Outcome Measures Using the GRADE Reporting Criteria

eFigure 1. PRISMA Flow Chart

eFigure 2. Effects of AID on Measures of Glucose Variability

eFigure 3. Risk of Bias Metrics for Randomized Control Trials Included in the Meta-Analyses

eFigure 4. Funnel Plots for Main Outcome Measures

eFigure 5. Effects of AID on Measures of Time in Range

This supplementary material has been provided by the authors to give readers additional information about their work.

© 2025 de Visser HS et al. *JAMA Pediatrics*.

eTable 1. Search Strategy and Results Medline

Database: Ovid MEDLINE(R)
Data range searched: January 1st 2017 to May 14th, 2024
Date searched: June 4th, 2024

1	adolescent/ or child/ or minors/ or exp puberty/ or adolescent health services/ or child health services/ or adolescent medicine/ or pediatrics/ or pediatric emergency medicine/ or adolescent health/ or child health/ or pediatricians/ or pediatric nurse practitioners/ or nurses, pediatric/ or pediatric nursing/ or pediatric assistants/ or hospitals, pediatric/ or intensive care units, pediatric/ (3260091)
2	(adolesc* or boy? or boyhood or girlhood or child* or girl? or juvenil* or kid? or minor? or stepchild* or paediatric* or peadiatric* or pediatric* or pubert* or pubescen* or prepubert* or prepubescen* or school* or highschool* or youngster* or preteen* or teen* or underage? or under-age? or youth*).ti,ab,kf. (2840992)
3	(pediatric* or paediatric* or child* or adolescen*).jn,jw. (770602)
4	(pediatric* or paediatric* or child* or adolescen*).in. (1473063)
5	or/1-4 (5360777)
6	Diabetes Mellitus/ or diabetes mellitus, type 1/ (229746)
7	(diabet* or IDDM or T1DM or T1D).ti,ab,kf. (823856)
8	or/6-7 (852095)
9	implantable insulin pump/ or insulin infusion/ or pancreas, artificial/ (16210)
10	((automat* or hybrid or smart or predictive or hack* or reverse engineer* or DIY or do-it-yourself) adj3 (insulin or glucose control* or glyc?emic control* or glucose management or glyc?emic management)).ti,ab,kf. (1670)
11	((artificial or automat* or bioartificial or bionic or robot* or predictive or diy or hack* or self* or yourself or reverse engineer*) adj2 (pancreas or bcell or b-cell or beta or betacell* or endocrine)).ti,ab,kf. (4235)
12	(loop* adj5 (diy or hack* or self* or yourself or reverse engineer*)).ti,ab,kf. (1303)
13	(close* loop or closedloop or closeloop or medtronic* or minimed or omnipod or camdiab or androidaps or android aps or dexcom or dana or tslim or camaps or openaps or open aps or controliq or control iq or basaliq or basal iq or looping or looper$1 or tidepool or diypancreas or diy pancreas or nightscout).ti,ab,kf. (28973)
14	or/9-13 (48064)
15	exp clinical trials as topic/ or random allocation/ or double-blind method/ or single-blind method/ or placebos/ (675450)
16	(clinical trial or adaptive clinical trial or clinical trial, phase I, or clinical trial, phase ii or clinical trial, phase iii or clinical trial, phase iv or controlled clinical trial or randomized controlled trial or equivalence trial or pragmatic clinical trial or multicenter study).pt. (1203836)
17	(clinical adj trial*).ti,ab,kf. (528777)
18	((singl* or doubl* or trebl* or tripl*) adj (blind* or mask* or dumm*)).ti,ab,kf. (205883)
19	(random* or placebo*).ti,ab,kf. (1614483)
20	or/15-19 (2743921)
21	5 and 8 and 14 and 20 (959)
22	limit 21 to (english language and yr="2017 -Current") (460)

© 2025 de Visser HS et al. *JAMA Pediatrics*.

eTable 2. Search Strategy and Results Embase

Database: Embase (Ovid)
Data range searched: January 1st 2017 to May 14th, 2024
Date searched: June 5th, 2024

1	exp adolescent/ or child/ or abandoned child/ or adopted child/ or boy/ or brain damaged child/ or child of impaired parents/ or disabled child/ or foster child/ or gifted child/ or girl/ or hospitalized child/ or institutionalized child/ or orphaned child/ or school child/ or single parent child/ or unwanted child/ or "minor (person)"/ or exp puberty/ or adolescent health/ or child health care/ or child hospitalization/ or pediatrics/ or pediatric emergency medicine/ or child urology/ or child health/ or pediatric patient/ or pediatrician/ or pediatric nurse practitioner/ or pediatric nurse/ or exp pediatric nursing/ or pediatric hospital/ (3400171)
2	(adolesc* or boy? or boyhood or girlhood or child* or girl? or juvenil* or kid? or minor? or stepchild* or paediatric* or peadiatric* or pediatric* or pubert* or pubescen* or prepubert* or prepubescen* or school* or highschool* or youngster* or preteen* or teen* or underage? or under-age? or youth*).ti,ab,kf. (3582574)
3	(pediatric* or paediatric* or child* or adolescen*).jn,jw. (953861)
4	(pediatric* or paediatric* or child* or adolescen*).in. (2263157)
5	or/1-4 (5990615)
6	Diabetes Mellitus/ or insulin dependent diabetes mellitus/ (858946)
7	(diabet* or IDDM or T1DM or T1D).ti,ab,kf. (1250073)
8	or/6-7 (1445548)
9	implantable insulin pump/ or insulin infusion/ or pancreas, artificial/) (12569)
10	((automat* or hybrid or smart or predictive or hack* or reverse engineer* or DIY or do-it-yourself) adj3 (insulin or glucose control* or glyc?emic control* or glucose management or glyc?emic management)).ti,ab,kf. (2902)
11	((artificial or automat* or bioartificial or bionic or robot* or predictive or diy or hack* or self* or yourself or reverse engineer*) adj2 (pancreas or bcell or b-cell or beta or betacell* or endocrine)).ti,ab,kf. (5885)
12	(loop* adj5 (diy or hack* or self* or yourself or reverse engineer*)).ti,ab,kf. (1450)
13	(close* loop or closedloop or closeloop or medtronic* or minimed or omnipod or camdiab or androidaps or android aps or dexcom or dana or tslim or camaps or openaps or open aps or controliq or control iq or basaliq or basal iq or looping or looper$1 or tidepool or diypancreas or diy pancreas or nightscout).ti,ab,kf. (47266)
14	or/9-13 (63890)
15	exp clinical trials as topic/ or random allocation/ or double-blind method/ or single-blind method/ or placebos/ (1071300)
16	(exp clinical trial/ (1910980)
17	(clinical adj trial*).ti,ab,kf. (765809)
18	((singl* or doubl* or trebl* or tripl*) adj (blind* or mask* or dumm*)).ti,ab,kf. (291744)
19	(random* or placebo*).ti,ab,kf. (2205057)
20	or/15-19 (3892090)
21	5 and 8 and 14 and 20 (1498)
22	limit 21 to (english language and yr="2017 -Current") (876)

© 2025 de Visser HS et al. *JAMA Pediatrics*.

eTable 3. Search Strategy and Results Cochrane

Database: COCHRANE Central (Ovid)
Data range searched: January 1st 2017 to May 14th, 2024
Date searched: June 5th, 2024

1	adolescent/ or child/ or minors/ or exp puberty/ or adolescent health services/ or child health services/ or adolescent medicine/ or pediatrics/ or pediatric emergency medicine/ or adolescent health/ or child health/ or pediatricians/ or pediatric nurse practitioners/ or nurses, pediatric/ or pediatric nursing/ or pediatric assistants/ or hospitals, pediatric/ or intensive care units, pediatric/ (170420)
2	(adolesc* or boy? or boyhood or girlhood or child* or girl? or juvenil* or kid? or minor? or stepchild* or paediatric* or peadiatric* or pediatric* or pubert* or pubescen* or prepubert* or prepubescen* or school* or highschool* or youngster* or preteen* or teen* or underage? or under-age? or youth*).ti,ab,kw. (259777)
3	(pediatric* or paediatric* or child* or adolescen*).jn,jw. (41369)
4	(pediatric* or paediatric* or child* or adolescen*).in. (37702)
5	or/1-4 (373367)
6	Diabetes Mellitus/ or diabetes mellitus, type 1/ (18668)
7	(diabet* or IDDM or T1DM or T1D).ti,ab,kf. (119709)
8	or/6-7 (120860)
9	exp infusion pumps/ or pancreas, artificial/ (1670)
10	((automat* or hybrid or smart or predictive or hack* or reverse engineer* or DIY or do-it-yourself) adj3 (insulin or glucose control* or glyc?emic control* or glucose management or glyc?emic management)).ti,ab,kw. (559)
11	((artificial or automat* or bioartificial or bionic or robot* or predictive or diy or hack* or self* or yourself or reverse engineer*) adj2 (pancreas or bcell or b-cell or beta or betacell* or endocrine)).ti,ab,kw. (633)
12	(loop* adj5 (diy or hack* or self* or yourself or reverse engineer*)).ti,ab,kw. (28)
13	(close* loop or closedloop or closeloop or medtronic* or minimed or omnipod or camdiab or androidaps or android aps or dexcom or dana or tslim or camaps or openaps or open aps or controliq or control iq or basaliq or basal iq or looping or looper$1 or tidepool or diypancreas or diy pancreas or nightscout).ti,ab,kw. (4472)
14	or/9-13 (6243)
15	5 and 8 and 14 (953)
16	limit 15 to (english language and yr="2017 -Current") (496)

© 2025 de Visser HS et al. *JAMA Pediatrics*.

eTable 4. Search Strategy and Results CINAHL

Database: CINAHL
Data range searched: January 1st 2017 to May 14th, 2024
Date searched: May 14th, 2024

1	(MH adolescence+) or (MH child) or (MH "child, abandoned") or (MH "child, adopted") or (MH "children with disabilities") or (MH "child, foster") or (MH "child, gifted") or (MH "child, hospitalized") or (MH "children of impaired parents+") or (MH "child, institutionalized") or (MH "children of LGBTQ+ persons") or (MH "child, medically fragile") or (MH "latchkey children") or (MH "only child") or (MH "minors (legal)") or (MH puberty+) or (MH "adolescent health services") or (MH "child health services") or (MH "adolescent medicine") or (MH pediatrics) or (MH "pediatric care") or (MH "adolescent health") or (MH "child health") or (MH pediatricians) or (MH "pediatric nurse practitioners+") or (MH "pediatric nurses") or (MH "pediatric critical care nurses+") or (MH "pediatric nursing") or (MH "pediatric critical care nursing+") or (MH "pediatric emergency nursing") or (MH "pediatric endocrinology nursing") or (MH "hospitals, pediatric") or (MH "pediatric units+") (975221)
2	(adolesc* or boy or boys or boyhood or girlhood or child* or girl or girls or juvenil* or kid or kids or minor or minors or stepchild* or paediatric* or peadiatric* or pediatric* or pubert* or pubescen* or prepubert* or prepubescen* or school* or highschool* or youngster* or preteen* or teen* or underage or underaged or "under age" or "under aged" or youth*) (1477243)
3	SO(pediatric* or paediatric* or child* or adolescen*) (377921)
4	AF(pediatric* or paediatric* or child* or adolescen*) (498124)
5	S1 or S2 or S3 or S4 (1714158)
6	(MH "diabetes mellitus") or (MH "diabetes mellitus, type 1") (100018)
7	(diabet* or IDDM or T1DM or T1D) (283106)
8	S6 or S7 (283106)
9	(MH "infusion pumps+") (6406)
10	((automat* or hybrid or smart or predictive or hack* or "reverse engineer*" or DIY or "do-it-yourself") N3 (insulin or "glucose control*" or "glyc#emic control*" or "glucose management" or "glyc#emic management")) (614)
11	((artificial or automat* or bioartificial or bionic or robot* or predictive or diy or hack* or self* or yourself or "reverse engineer*") N2 (pancreas or bcell or "b-cell" or beta or betacell* or endocrine)) (702)
12	(loop* N5 (diy or hack* or self* or yourself or "reverse engineer*")) (109)
13	("close* loop" or closedloop or closeloop or medtronic* or minimed or omnipod or camdiab or androidaps or "android aps" or dexcom or dana or tslim or camaps or openaps or "open aps" or controliq or "control iq" or basaliq or "basal iq" or looping or looper or loopers or tidepool or diypancreas or "diy pancreas" or nightscout) (4643)
14	S9 or S10 or S11 or S12 or S13 (11293)
15	(MH "clinical trials+") or (MH "random assignment") or (MH placebos) (380634)
16	(ZT "clinical trial") (112968)
17	(clinical N1 trial*) (298819)
18	((singl* or doubl* or trebl* or tripl*) N1 (blind* or mask* or dumm*)) (89036)
19	(random* or placebo*) (546470)
20	S15 or S16 or S17 or S18 or S19 (721728)
21	S5 and S8 and S14 and S20 (432)
22	S21 Limiters - Publication Date: 20170101-; English Language (222)

© 2025 de Visser HS et al. *JAMA Pediatrics*.

eTable 5. Search Strategy and Results Medline (to March 11 2025)

adolescent/ or child/ or minors/ or exp puberty/ or adolescent health services/ or child health services/ or adolescent medicine/ or pediatrics/ or pediatric emergency medicine/ or adolescent health/ or child health/ or pediatricians/ or pediatric nurse practitioners/ or nurses, pediatric/ or pediatric nursing/ or pediatric assistants/ or hospitals, pediatric/ or intensive care units, pediatric/	3334681
(adolesc* or boy? or boyhood or girlhood or child* or girl? or juvenil* or kid? or minor? or stepchild* or paediatric* or peadiatric* or pediatric* or pubert* or pubescen* or prepubert* or prepubescen* or school* or highschool* or youngster* or preteen* or teen* or underage? or under-age? or youth*).	2946922
(pediatric* or paediatric* or child* or adolescen*).jn,jw.	793354
(pediatric* or paediatric* or child* or adolescen*).in.	1562217
or/1-4	5544138
Diabetes Mellitus/ or diabetes mellitus, type 1/	234234
(diabet* or IDDM or T1DM or T1D).	862641
or/6-7	890917
implantable insulin pump/ or insulin infusion/ or pancreas, artificial/	1068
((automat* or hybrid or smart or predictive or hack* or reverse engineer* or DIY or do-it-yourself) adj3 (insulin or glucose control* or glyc?emic control* or glucose management or glyc?emic management)).ti,ab,kf.	1906
((artificial or automat* or bioartificial or bionic or robot* or predictive or diy or hack* or self* or yourself or reverse engineer*) adj2 (pancreas or bcell or b-cell or beta or betacell* or endocrine)). 4445 (loop* adj5 (diy or hack* or self* or yourself or reverse engineer*)).ti,ab,kf.	1381
(close* loop or closedloop or closeloop or medtronic* or minimed or omnipod or camdiab or androidaps or android aps or dexcom or dana or tslim or camaps or openaps or open aps or controliq or control iq or basaliq or basal iq or looping or looper$1 or tidepool or diypancreas or diy pancreas or nightscout).	30799
or/9-13	36811
exp clinical trials as topic/ or random allocation/ or double-blind method/ or single-blind method/ or placebos/	692110
(clinical trial or adaptive clinical trial or clinical trial, phase I, or clinical trial, phase ii or clinical trial, phase iii or clinical trial, phase iv or controlled clinical trial or randomized controlled trial or equivalence trial or pragmatic clinical trial or multicenter study).	1238431
(clinical adj trial*).	560693
((singl* or doubl* or trebl* or tripl*) adj (blind* or mask* or dumm*)).ti,ab,kf.	212444
(random* or placebo*).	1699070
or/15-19	2862335
5 and 8 and 14 and 20	602
limit 21 to (english language and yr="2024 -Current")	89

© 2025 de Visser HS et al. *JAMA Pediatrics*.

eTable 6. Search Strategy and Results Embase (to March 11 2025)

exp adolescent/ or child/ or abandoned child/ or adopted child/ or boy/ or brain damaged child/ or child of impaired parents/ or disabled child/ or foster child/ or gifted child/ or girl/ or hospitalized child/ or institutionalized child/ or orphaned child/ or school child/ or single parent child/ or unwanted child/ or "minor (person)"/ or exp puberty/ or adolescent health/ or child health care/ or child hospitalization/ or pediatrics/ or pediatric emergency medicine/ or child urology/ or child health/ or pediatric patient/ or pediatrician/ or pediatric nurse practitioner/ or pediatric nurse/ or exp pediatric nursing/ or pediatric hospital/	3522962
(adolesc* or boy? or boyhood or girlhood or child* or girl? or juvenil* or kid? or minor? or stepchild* or paediatric* or peadiatric* or pediatric* or pubert* or pubescen* or prepubert* or prepubescen* or school* or highschool* or youngster* or preteen* or teen* or underage? or under-age? or youth*).ti,ab,kf	3708347
(pediatric* or paediatric* or child* or adolescen*).jn,jw.	982357
(pediatric* or paediatric* or child* or adolescen*).in.	2361639
or/1-4	6201716
Diabetes Mellitus/ or insulin dependent diabetes mellitus/	901080
(diabet* or IDDM or T1DM or T1D).ti,ab,kf.	1302567
or/6-7	1513329
implantable insulin pump/ or insulin infusion/ or pancreas, artificial/	12847
((automat* or hybrid or smart or predictive or hack* or reverse engineer* or DIY or do-it-yourself) adj3 (insulin or glucose control* or glyc?emic control* or glucose management or glyc?emic management)).ti,ab,kf.	3240
((artificial or automat* or bioartificial or bionic or robot* or predictive or diy or hack* or self* or yourself or reverse engineer*) adj2 (pancreas or bcell or b-cell or beta or betacell* or endocrine)).ti,ab,kf. 6145 (loop* adj5 (diy or hack* or self* or yourself or reverse engineer*)).ti,ab,kf.	1526
(close* loop or closedloop or closeloop or medtronic* or minimed or omnipod or camdiab or androidaps or android aps or dexcom or dana or tslim or camaps or openaps or open aps or controliq or control iq or basaliq or basal iq or looping or looper$1 or tidepool or diypancreas or diy pancreas or nightscout).ti,ab,kf.	50649
or/9-13	67944
exp "clinical trial (topic)"/ or randomization/ or double blind procedure/ or single blind procedure/ or triple blind procedure/ or placebo/	1107817
exp clinical trial/	1983574
(clinical adj trial*).ti,ab,kf.	806610
((singl* or doubl* or trebl* or tripl*) adj (blind* or mask* or dumm*)).ti,ab,kf.	300543
(random* or placebo*).ti,ab,kf.	2305106
or/15-19	4051440
5 and 8 and 14 and 20	1598
limit 21 to (english language and yr="2024 -Current")	149

© 2025 de Visser HS et al. *JAMA Pediatrics*.

eTable 7. Search Strategy and Results Cochrane (to March 11 2025)

adolescent/ or child/ or minors/ or exp puberty/ or adolescent health services/ or child health services/ or adolescent medicine/ or pediatrics/ or pediatric emergency medicine/ or adolescent health/ or child health/ or pediatricians/ or pediatric nurse practitioners/ or nurses, pediatric/ or pediatric nursing/ or pediatric assistants/ or hospitals, pediatric/ or intensive care units, pediatric/	168468
(adolesc* or boy? or boyhood or girlhood or child* or girl? or juvenil* or kid? or minor? or stepchild* or paediatric* or peadiatric* or pediatric* or pubert* or pubescen* or prepubert* or prepubescen* or school* or highschool* or youngster* or preteen* or teen* or underage? or under-age? or youth*).ti,ab,kw.	268617
(pediatric* or paediatric* or child* or adolescen*).jn,jw.	41269
(pediatric* or paediatric* or child* or adolescen*).in.	36920
or/1-4	380758
Diabetes Mellitus/ or diabetes mellitus, type 1/	18848
(diabet* or IDDM or T1DM or T1D).ti,ab,kw.	122963
or/6-7	124121
exp infusion pumps/ or pancreas, artificial/	1668
((automat* or hybrid or smart or predictive or hack* or reverse engineer* or DIY or do-it-yourself) adj3 (insulin or glucose control* or glyc?emic control* or glucose management or glyc?emic management)).ti,ab,kw.	567
((artificial or automat* or bioartificial or bionic or robot* or predictive or diy or hack* or self* or yourself or reverse engineer*) adj2 (pancreas or bcell or b-cell or beta or betacell* or endocrine)).ti,ab,kw.	646
(loop* adj5 (diy or hack* or self* or yourself or reverse engineer*)).ti,ab,kw.	30
(close* loop or closedloop or closeloop or medtronic* or minimed or omnipod or camdiab or androidaps or android aps or dexcom or dana or tslim or camaps or openaps or open aps or controliq or control iq or basaliq or basal iq or looping or looper$1 or tidepool or diypancreas or diy pancreas or nightscout).ti,ab,kw.	4693
or/9-13	6463
5 and 8 and 14	967
limit 15 to (english language and yr="2024 -Current")	53

© 2025 de Visser HS et al. *JAMA Pediatrics*.

eTable 8. Search Strategy and Results CINAHL (to March 11 2025)

(MH adolescence+) or (MH child) or (MH "child, abandoned") or (MH "child, adopted") or (MH "children with disabilities") or (MH "child, foster") or (MH "child, gifted") or (MH "child, hospitalized") or (MH "children of impaired parents+") or (MH "child, institutionalized") or (MH "children of LGBTQ+ persons") or (MH "child, medically fragile") or (MH "latchkey children") or (MH "only child") or (MH "minors (legal)") or (MH puberty+) or (MH "adolescent health services") or (MH "child health services") or (MH "adolescent medicine") or (MH pediatrics) or (MH "pediatric care") or (MH "adolescent health") or (MH "child health") or (MH pediatricians) or (MH "pediatric nurse practitioners+") or (MH "pediatric nurses") or (MH "pediatric critical care nurses+") or (MH "pediatric nursing") or (MH "pediatric critical care nursing+") or (MH "pediatric emergency nursing") or (MH "pediatric endocrinology nursing") or (MH "hospitals, pediatric") or (MH "pediatric units+")	999385
(adolesc* or boy or boys or boyhood or girlhood or child* or girl or girls or juvenil* or kid or kids or minor or minors or stepchild* or paediatric* or peadiatric* or pediatric* or pubert* or pubescen* or prepubert* or prepubescen* or school* or highschool* or youngster* or preteen* or teen* or underage or underaged or "under age" or "under aged" or youth*)	1526701
SO(pediatric* or paediatric* or child* or adolescen*)	395305
AF(pediatric* or paediatric* or child* or adolescen*)	529420
S1 or S2 or S3 or S4	1781576
(MH "diabetes mellitus") or (MH "diabetes mellitus, type 1")	101896
(diabet* or IDDM or T1DM or T1D)	293000
S6 or S7	293000
(MH "infusion pumps+")	6493
((automat* or hybrid or smart or predictive or hack* or "reverse engineer*" or DIY or "do-it-yourself") N3 (insulin or "glucose control*" or "glyc#emic control*" or "glucose management" or "glyc#emic management"))	682
((artificial or automat* or bioartificial or bionic or robot* or predictive or diy or hack* or self* or yourself or "reverse engineer*") N2 (pancreas or bcell or "b-cell" or beta or betacell* or endocrine))	738
(loop* N5 (diy or hack* or self* or yourself or "reverse engineer*"))	117
("close* loop" or closedloop or closeloop or medtronic* or minimed or omnipod or camdiab or androidaps or "android aps" or dexcom or dana or tslim or camaps or openaps or "open aps" or controliq or "control iq" or basaliq or "basal iq" or looping or looper or loopers or tidepool or diypancreas or "diy pancreas" or nightscout)	4826
S9 or S10 or S11 or S12 or S13	11611
(MH "clinical trials+") or (MH "random assignment") or (MH placebos)	387072
(ZT "clinical trial")	114489
(clinical N1 trial*)	305221
((singl* or doubl* or trebl* or tripl*) N1 (blind* or mask* or dumm*))	90779
(random* or placebo*)	566066
S15 or S16 or S17 or S18 or S19	744421

© 2025 de Visser HS et al. *JAMA Pediatrics*.

eTable 9. Baseline Characteristics of Participants in AID Group vs Standard Diabetes Management included in the meta-analysis.

	AID Systems	Control
Mean age	12.6 ± 2.4	12.7 ± 2.3
Female sex (%)	52	44
Mean Duration of Diabetes (yrs)[a]	5.8 ± 3.4	6.5 ± 3.3
Race or ethnic group (%)		
White, non-Hispanic	79	81
Hispanic or Latino	5	6
Black	4	3
Asian	3.4	3
American Indian or Alaskan Native	0.3	0.1
Maori	19	3
Multiracial	6	5
Missing	0.6	0.1
Highest parent education level (%)[b]		
< Bachelor's degree	31	25
Bachelor's degree	33	33
Graduate or professional degree	34	41
Annual household income (%)[c]		
<$100,000	36	33
≥$100,000	63	64
Mean glycated hemoglobin level at screening[d]	8.5 ± 0.1	8.4 ± 0.9
Mean time in range at screening[d]	51.0 ± 14.3	51.7 ± 13.7

[a]Information only reported by trials Ware et al., 2022, Breton et al., 2020, and Messer et al., 2022
[b]Information reported by all trials except Reiss et al., 2022, Boughton et al., 2022, Abraham et al., 2021, Brown et al., 2019, Tauschmann et al., 2018, and Garg et al., 2023.
[c]Information only reported by trials Burnside et al., 2022, Breton et al., 2020, Messer et al., 2022, and McVean et al., 2023.
[d]Information reported by all trials except Tauschmann et al., 2018.

© 2025 de Visser HS et al. *JAMA Pediatrics*.

eTable 10. Safety outcomes reported in participants included in the meta-analysis.

	AID systems	Control
Total events*	77	41
Severe hypoglycemia[a]	2	1
DKA[a]	1	0
Hyperglycemia without DKA related to pump*	20	0
Other events*	27	22

*Information reported by all trials except Abraham et al., 2021, Brown et al., 2019, Tauschmann et al., 2018, and Garg et al., 2023.
[a]Information reported by all trials except Brown et al., 2019, Tauschmann et al., 2018, and Garg et al., 2023.

© 2025 de Visser HS et al. *JAMA Pediatrics*.

eTable 11. Certainty of evidence for observed changes in outcome measures using the GRADE Reporting criteria

**Outcome**	**MID**	**Observed effect**	**Bias**	**Imprecision**	**Incon**	**Indirect**	**Publ Bias**
Measures of glucose management							
Time in range	5%	11.5% (9.3 to 13.7%)	Low	Low	Low	Medium	Low
HbA1c	0.5%	0.41% (−0.58 to −0.25%)	Low	Low	Medium	Medium	Medium
Time in hyperglycemia	5%	−10.8% (–14.4 to −7.2%)	Low	Low	Low	Medium	Low
Time in hypoglycemia	1%	−0.32% (–0.60 to −0.03%)	Low	Low	Medium	Medium	Low
Day time glucose management							
Time in range	2.5%	+8.5%(5.9 to 11.1%)	Medium	Low	Low	Medium	Low
Time in hyperglycemia	−2.5%	−5.6%(−8.4 to −2.8%)	Medium	Low	Low	Medium	Low
Time in hypoglycemia	0.5%	−0.30%(−0.72 to +0.12%)	Medium	Low	High	Medium	Low
Night time glucose management							
Time in range	2.5%	+19.7% (17.0 to 22.4%)	Medium	Low	Low	Medium	Low
Time in hyperglycemia	2.5%	−14.4% (−19.9 to −8.9%)	Medium	Low	Low	Medium	Low
Time in hypoglycemia	0.5%	−0.62% (−1.02% to −0.23%)	Medium	Low	High	Medium	Low
Adverse events							
Any event	NA	0.72 (0.27 to 1.92)	Low	High	High	Medium	Low
Severe hypoglycemia	NA	1.70 (0.63 to 4.60)	Low	High	Medium	Medium	Low
Diabetic Ketoacidosis	NA	2.24 (1.13 to 4.42)	Low	High	Low	Medium	Low

© 2025 de Visser HS et al. *JAMA Pediatrics*.

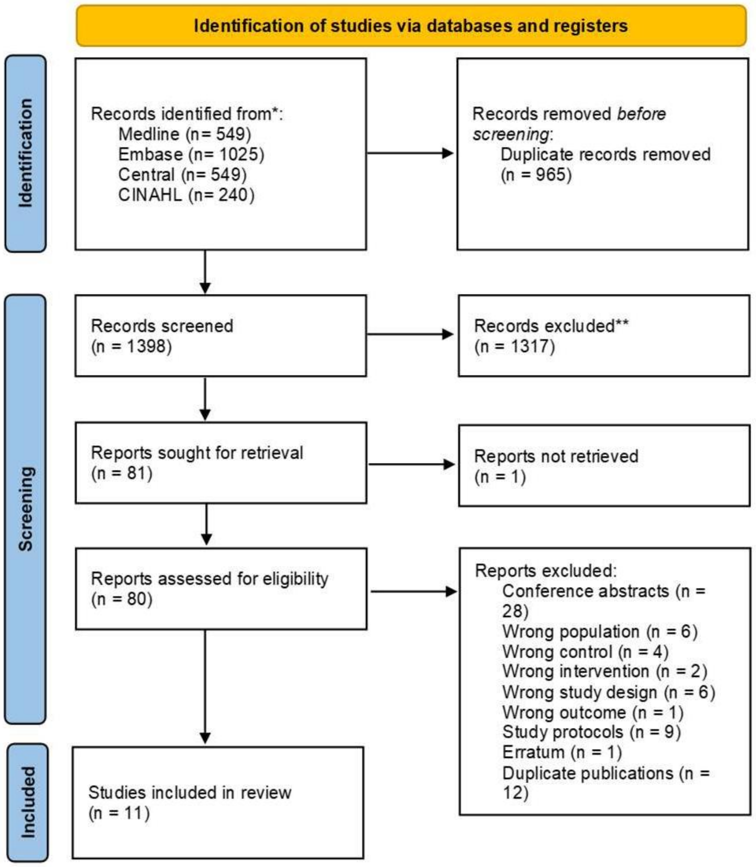

*Consider, if feasible to do so, reporting the number of records identified from each database or register searched (rather than the total number across all databases/registers).

**If automation tools were used, indicate how many records were excluded by a human and how many were excluded by automation tools.

From: Page MJ, McKenzie JE, Bossuyt PM, Boutron I, Hoffmann TC, Mulrow CD, et al. The PRISMA 2020 statement: an updated guideline for reporting systematic reviews. BMJ 2021;372:n71. doi: 10.1136/bmj.n71

eFigure 1. PRISMA Flow chart

© 2025 de Visser HS et al. *JAMA Pediatrics*.

A- Glucose Standard Deviation(SD)

Study(ref)	Experimental Mean	SD	Total	Control Mean	SD	Total	Weight	Mean Difference IV, Random, 95% CI
Garg lowA1c 2023(43)	58.80	10.5000	7	71.70	15.4000	8	5.3%	−12.90 [−26.11; 0.31]
Breton 2020(8)	61.00	11.0000	78	70.00	13.0000	22	13.4%	−9.00 [−14.96; −3.04]
Messer 2022(45)	66.00	11.0000	112	73.00	15.0000	53	16.1%	−7.00 [−11.52; −2.48]
Burnside 2022(42)	62.20	10.6000	20	67.60	13.6000	27	11.8%	−5.40 [−12.32; 1.52]
Abraham 2021(41)	64.00	13.0000	58	69.00	14.0000	53	15.1%	−5.00 [−10.04; 0.04]
Boughton 2022(7)	64.00	16.0000	44	67.00	25.0000	33	8.0%	−3.00 [−12.75; 6.75]
Ware 2022(29)	3.90	0.8000	19	4.30	1.0000	24	22.1%	−0.40 [−0.94; 0.14]
Garg highA1c 2023(43)	71.70	9.5000	21	71.00	21.4000	23	8.2%	0.70 [−8.94; 10.34]
**Total (95% CI)**			**359**			**243**	**100.0%**	**−4.68 [−8.14; −1.22]**

Heterogeneity: $Tau^2 = 14.0273$; $Chi^2 = 24.23$, df = 7 (P = 0.0010); $I^2 = 71.1\%$

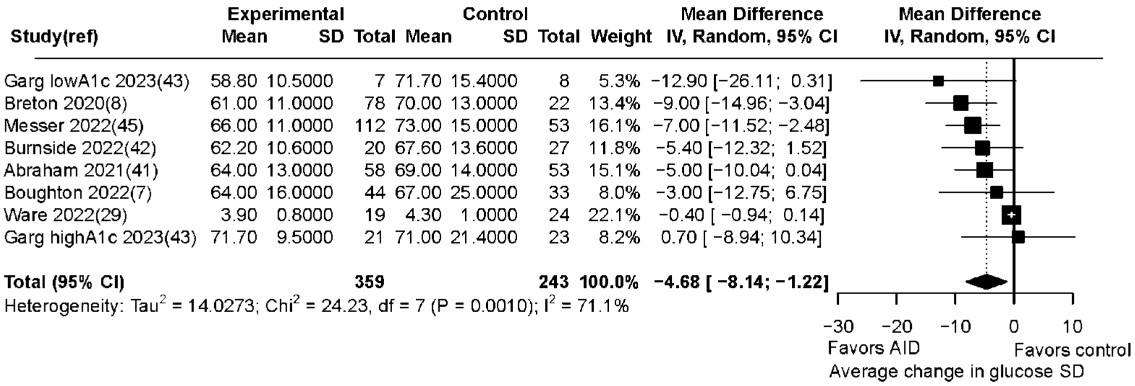

B- Glucose Coefficient of Variation (COV)

Study(ref)	Experimental Mean	SD	Total	Control Mean	SD	Total	Weight	Mean Difference IV, Random, 95% CI
Reiss 2022(46)	−2.31	1.4082	21	1.29	1.1071	21	16.4%	−3.60 [−4.37; −2.83]
Garg low A1c 2023(43)	37.20	5.8000	7	40.60	6.5000	8	5.6%	−3.40 [−9.62; 2.82]
Abraham 2021(41)	38.35	5.3111	58	41.00	6.0929	53	13.6%	−2.65 [−4.78; −0.51]
Messer 2022(45)	38.00	4.0000	112	39.00	5.0000	53	15.1%	−1.00 [−2.54; 0.54]
Breton 2020(8)	38.00	4.0000	78	39.00	4.0000	22	14.2%	−1.00 [−2.89; 0.89]
Burnside 2022(42)	39.70	5.2000	20	37.40	5.9000	27	11.0%	2.30 [−0.89; 5.49]
Garg high A1c 2023(43)	41.20	3.4000	21	38.50	13.1000	23	6.5%	2.70 [−2.85; 8.25]
Boughton 2022(7)	42.00	7.0000	44	39.00	8.0000	33	10.5%	3.00 [−0.42; 6.42]
Ware 2022(29)	49.00	8.0000	19	45.00	9.0000	24	7.2%	4.00 [−1.09; 9.09]
**Total (95% CI)**			**380**			**264**	**100.0%**	**−0.41 [−2.20; 1.38]**

Heterogeneity: $Tau^2 = 4.9358$; $Chi^2 = 42.05$, df = 8 (P < 0.0001); $I^2 = 81.0\%$

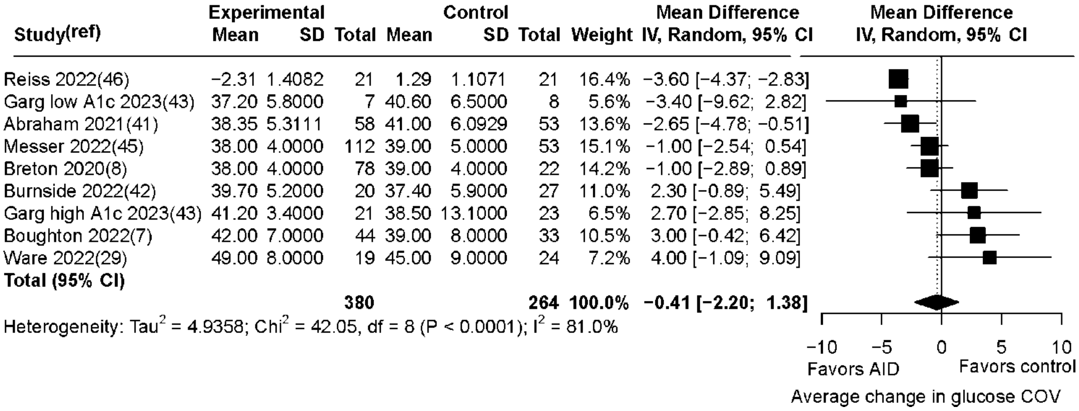

eFigure 2. Effects of automated insulin delivery systems on measures of glucose variability

© 2025 de Visser HS et al. *JAMA Pediatrics*.

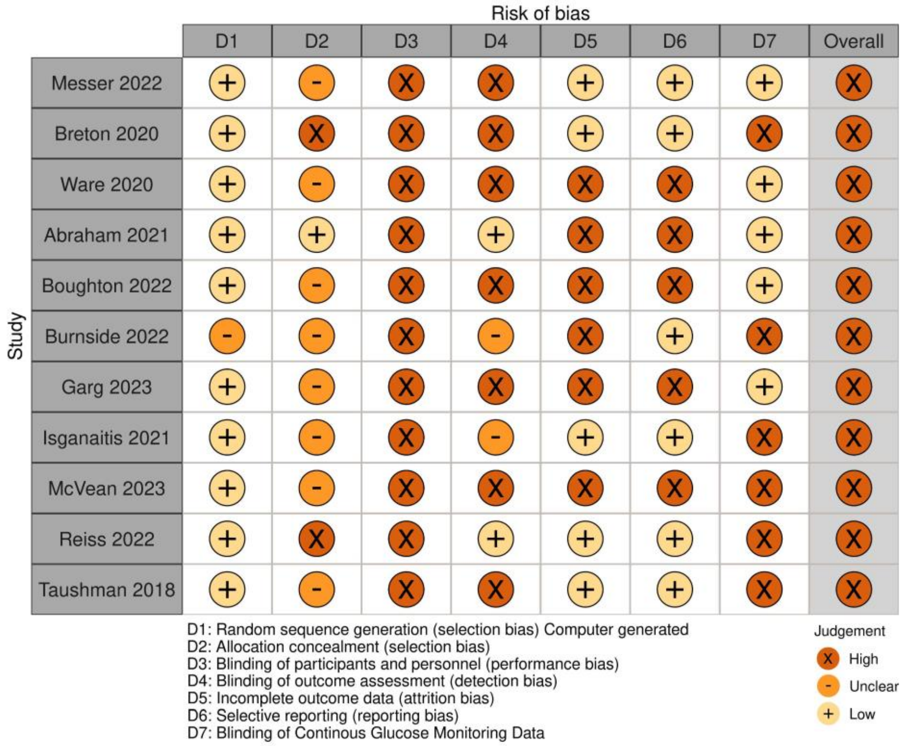

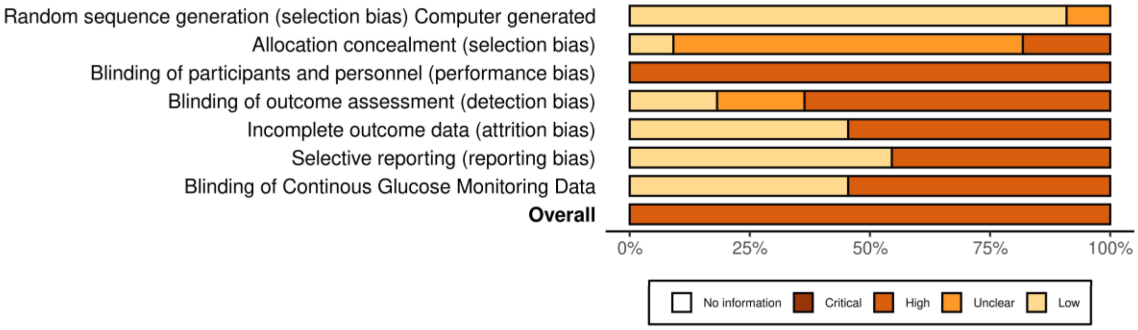

eFigure 3. Risk of bias metrics for randomized control trials included in the meta-analyses

© 2025 de Visser HS et al. *JAMA Pediatrics*.

A – Time in Range 3.9-10.0 mmol/L

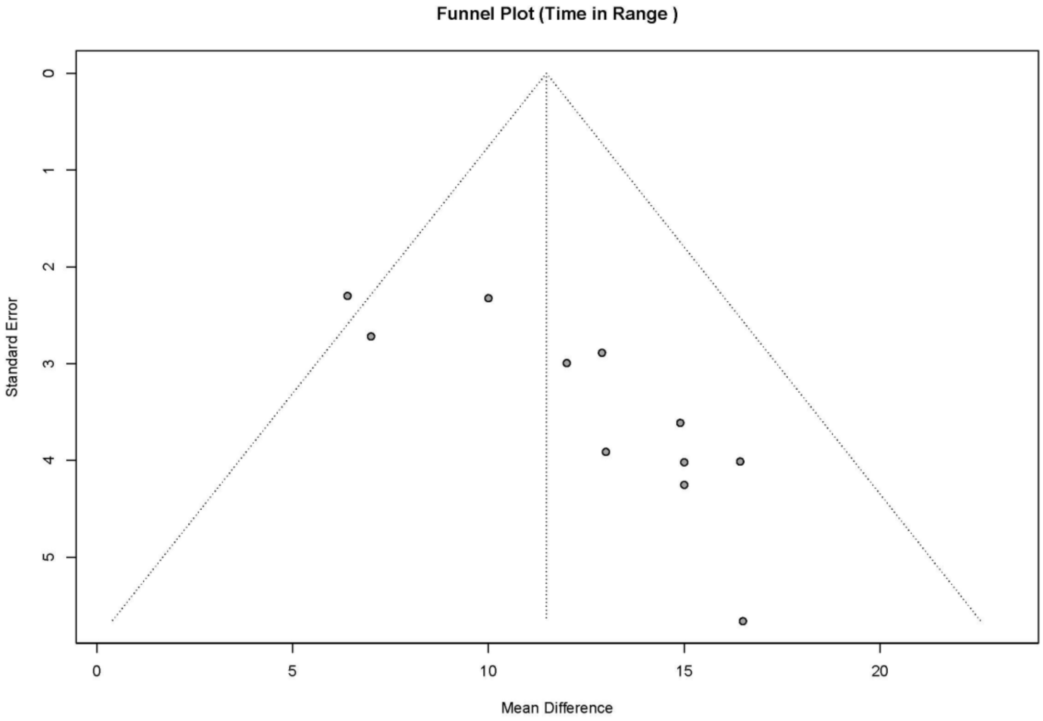

B – HbA1c

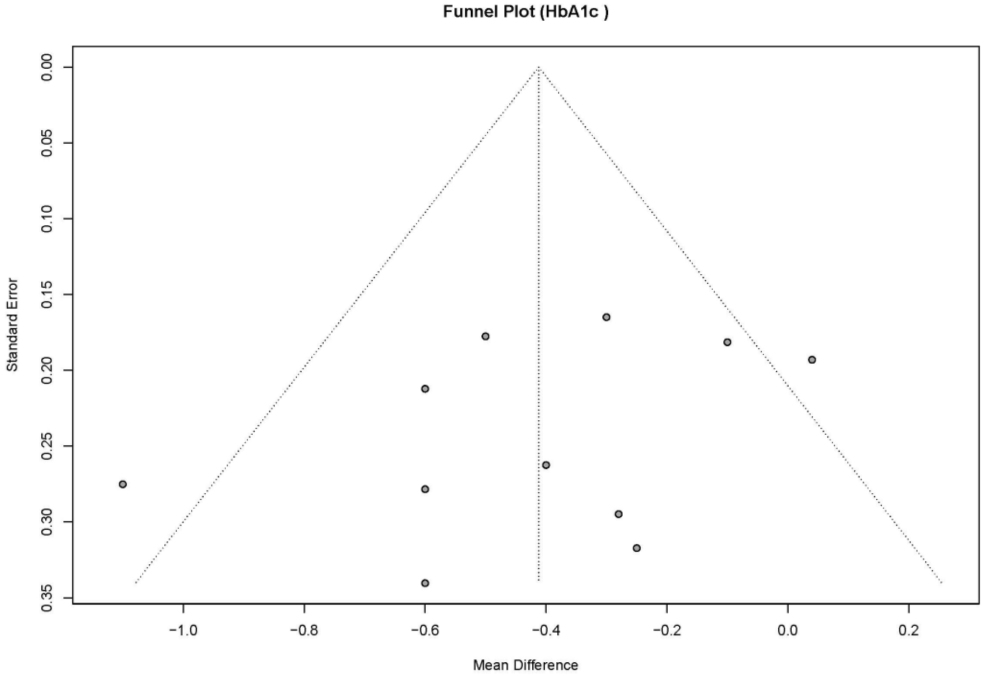

eFigure 4. Funnel plots for main outcome measures.

© 2025 de Visser HS et al. *JAMA Pediatrics*.

eFigure 5. Effects of AID on measures of time in range

Time in hypoglycemia

Study(ref) or Subgroup	Experimental Mean	SD	Total	Control Mean	SD	Total	Weight	Mean Difference IV, Random, 95% CI
**Time = day**								
Breton 2020(8)	1.70	1.5008	78	2.17	1.5934	22	13.0%	−0.47 [−1.21; 0.27]
Messer 2022(45)	1.91	1.4264	112	2.17	1.8279	53	23.1%	−0.27 [−0.82; 0.29]
Burnside 2022(42)	2.40	1.5000	20	2.60	2.8000	27	4.7%	−0.20 [−1.44; 1.04]
Ware 2022(29)	7.12	5.0794	19	5.52	8.1633	24	0.5%	1.60 [−2.38; 5.59]
**Total (95% CI)**			**229**			**126**	**41.2%**	**−0.30 [−0.72; 0.12]**
Heterogeneity: $Tau^2 = 0$; $Chi^2 = 1.11$, df = 3 (P = 0.7738); $I^2 = 0\%$								
**Time = night**								
Burnside 2022(42)	1.20	1.7000	20	3.10	3.5000	27	3.1%	−1.90 [−3.42; −0.38]
Boughton 2022(7)	0.90	0.9000	42	1.50	1.5000	33	21.4%	−0.60 [−1.18; −0.02]
Messer 2022(45)	1.64	1.4264	112	2.18	1.9802	53	20.3%	−0.55 [−1.14; 0.05]
Breton 2020(8)	1.07	1.0407	78	1.55	1.6405	22	13.8%	−0.48 [−1.21; 0.24]
Ware 2022(29)	9.85	12.0635	19	5.99	10.1256	24	0.2%	3.87 [−2.90; 10.64]
**Total (95% CI)**			**271**			**159**	**58.8%**	**−0.62 [−1.02; −0.23]**
Heterogeneity: $Tau^2 = 0.0287$; $Chi^2 = 4.62$, df = 4 (P = 0.3284); $I^2 = 13.4\%$								
**Total (95% CI)**			**500**			**285**	**100.0%**	**−0.48 [−0.75; −0.22]**
Heterogeneity: $Tau^2 = 0$; $Chi^2 = 6.98$, df = 8 (P = 0.5389); $I^2 = 0.0\%$								
Test for subgroup differences: $Chi^2 = 1.20$, df = 1 (P = 0.2739)								

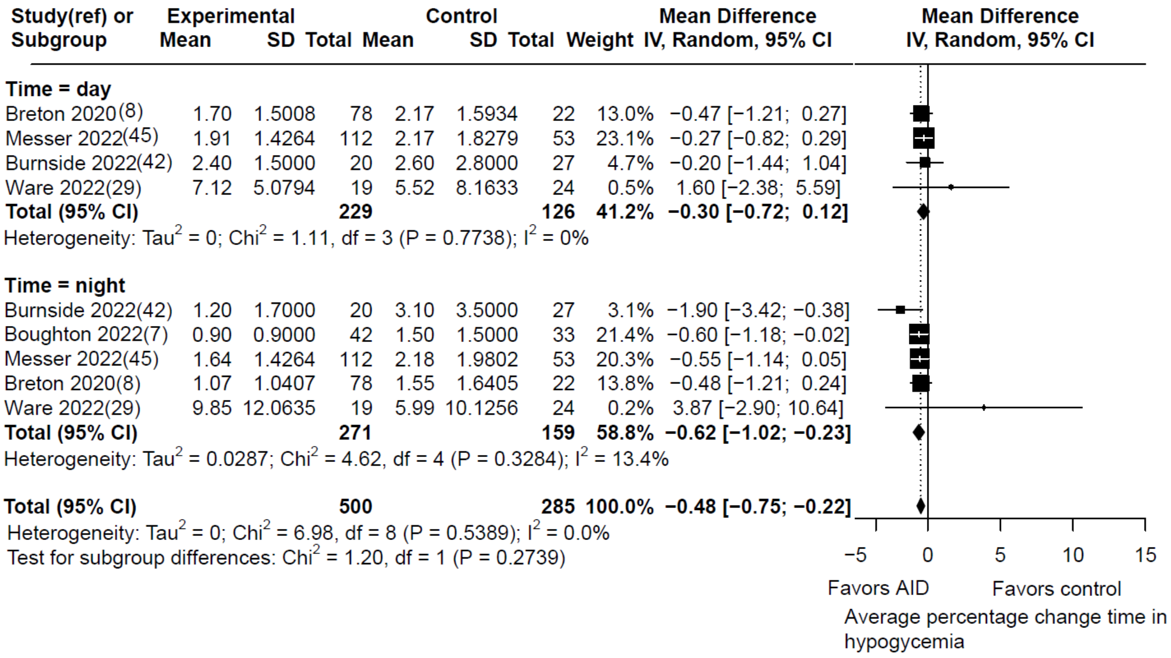

© 2025 de Visser HS et al. *JAMA Pediatrics*.
