# Supplementary material for: Automated Insulin Delivery Systems and Glucose Management in Children and Adolescents With Type 1 Diabetes: A Systematic Review and Meta-Analysis
Source: JAMA Pediatr. 2025 Sep 8;179(11):1162–71. doi: 10.1001/jamapediatrics.2025.2740 (PMC12418225; doi:10.1001/jamapediatrics.2025.2740)
Supplement: Supplement 2. — Nonauthor Collaborators. TEAM Trial Patient Coresearchers [file jamapediatr-e252740-s002.pdf]

Supplemental Online Content: Nonauthor Collaborators

*Indicates required information. Only first name, last name, and suffix will appear in PubMed.

***Group Name(s): TEAM Trial Patient Coresearchers**								
***First Name and Middle Initial(s)**	***Last Name**	***Suffix (eg, Jr, III)**	Academic Degrees	Institution	Location (city, state/province, country)	Role or Contribution, eg, chair, principal investigator	Group (if more than 1 Group listed in the byline) and/or Subgroup (eg, Steering Committee)	
Dana	Greenberg			Diabetes Action Canada	Toronto, ON	Patient partner	TEAM trial patient co-researchers	
Marley	Greenberg		MA	Diabetes Action Canada	Toronto, ON	Patient partner	TEAM trial patient co-researchers	
Ethan	Parikh		BSc	Diabetes Action Canada	Toronto, ON	Patient partner	TEAM trial patient co-researchers	
Cameron	Keighron		PhD	T1DNow	Galway, Ireland	Patient partner	TEAM trial patient co-researchers	
Bretton	Tyler			Diabetes Action Canada	Winnipeg, Manitoba	Patient partner	TEAM trial patient co-researchers	
Laura	Nemi		BA	Diabetes Action Canada	Mississauga, ON	Patient partner	TEAM trial patient co-researchers	
