# Supplementary material for: Automated Insulin Delivery Systems and Glucose Management in Children and Adolescents With Type 1 Diabetes: A Systematic Review and Meta-Analysis
Source: JAMA Pediatr. 2025 Sep 8;179(11):1162–71. doi: 10.1001/jamapediatrics.2025.2740 (PMC12418225; doi:10.1001/jamapediatrics.2025.2740)
Supplement: Supplement 3. — Data Sharing Statement. [file jamapediatr-e252740-s003.pdf]

Data Sharing Statement

de Visser. Automated Insulin Delivery Systems and Glucose Management in Children and Adolescents With Type 1 Diabetes. *JAMA Pediatr*. Published September 08, 2025. doi:10.1001/jamapediatrics.2025.2740

Data
Data available: Yes
Data types: Deidentified participant data
How to access data: E-mail corresponding author
When available: With publication

Supporting Documents
Document types: Statistical/analytic code
How to access documents: Attached in appendix
When available: With publication

Additional Information
Who can access the data: anyone requesting the data
Types of analyses: for any purpose
Mechanisms of data availability: after approval of a proposal
